# Physicochemical Characteristics of Porous Starch Obtained by Combined Physical and Enzymatic Methods—Part 2: Potential Application as a Carrier of Gallic Acid

**DOI:** 10.3390/molecules29153570

**Published:** 2024-07-29

**Authors:** Agnieszka Ewa Wiącek, Monika Sujka

**Affiliations:** 1 Department of Interfacial Phenomena, Faculty of Chemistry, Maria Curie-Skłodowska University, Maria Curie-Skłodowska Sq.3, 20-031 Lublin, Poland; 2 Department of Analysis and Food Quality Assessment, Faculty of Food Sciences and Biotechnology, University of Life Sciences in Lublin, Skromna St. 8, 20-704 Lublin, Poland

**Keywords:** starch, Washburn equation, surface and energetic parameters, polyphenol carrier

## Abstract

Wettability measurements were performed for aqueous dispersions of native and modified corn, potato, and pea starch granules deposited on glass plates by the thin layer method using test liquids of a different chemical nature (polar water and formamide or non-polar diiodomethane). High values of the determination coefficient *R*^2^ confirm that the linear regression model describes the relationship between the wetting time and the square of the penetration distance very well, indicating the linear nature of the Washburn relationship. A change in free energy (enthalpy) during the movement of the liquid in the porous layer was determined for all starches before and after modification in contact with test liquids. Wetting times for polar liquids increased significantly (from 3 to 4 fold), especially for corn starch. The lower the value of the adhesive tension, the easier the wetting process takes place, and consequently, the adsorption process is facilitated. Adhesive tension for polar substances applies to the adsorption of hydrophilic substances, while in the case of apolar substances, adhesive tension applies to the adsorption of hydrophobic substances. For the adsorption of gallic acid on starch, the relationships obtained for polar substances are crucial. The adsorption of gallic acid by forming hydrogen bonds or, more generally, donor–acceptor (acid–base) bonds is definitely higher for corn starch than other starches. Therefore, this starch has the most significant potential for use as a carrier of gallic acid or, more broadly, compounds from the polyphenol group.

## 1. Introduction

Polyphenols are a group of phytochemicals, which are responsible for the sensory attributes of plants, vegetables, and fruits, such as color, flavor, astringency, and smell, but above all, they demonstrate strong antioxidant properties. The most common division of polyphenols is into flavonoids (such as flavanones, flavans, flavonols, anthocyanidins, proanthocyanidins, dihydro flavonols, isoflavones, flavan-3-ols) and non-flavonoids (among others benzoic and cinnamic acids, xanthones, stilbenes, chalcones, lignans, benzophenones, coumarins, simple phenols, hydrolyzable tannins). One of the representatives of the latter group is gallic acid (GA; 3,4,5-trihydroxybenzoic acid) [1,2]. 

GA and its derivatives show a number of pro-healthy properties: anticarcinogenic [3,4], anti-inflammatory [5], antimicrobial [4,6], antiviral [7], antiallergic [8], neuroprotective [9], and hepatoprotective [5]. However, the potential use of gallic acid as an ingredient in medicines or functional foods is limited due to its astringent taste and relatively poor solubility, bioavailability, and stability [10,11]. The encapsulation technique can help overcome this problem by increasing the water solubility of GA and protecting this phytochemical against unfavorable external conditions [12,13]. Starch, including native and modified starch granules, can be used as a carrier of polyphenols [14,15,16]. To increase polyphenol encapsulation, microporous starch, starch nanoparticles, substituted starches, cross-linked starch, hydrolyzed starch, amylose inclusion complexes, and other forms of modified starch are developed [17,18,19,20]. Taking into account the beneficial properties of gallic acid mentioned above, starch loaded with this compound can be used in the food industry for the production of functional foods (e.g., for consumers with metabolic disorders) and active food packaging. 

Adsorption is the most common method used for the stabilization of different substances in starch, and it is a method very often used in the encapsulation process. In this process, an adsorbate (gas, liquid, or solute) interacts with the surface of the adsorbent both by physical (physisorption) and chemical (chemisorption) forces. These interactions between the adsorbate and adsorbent consist of weak chemical bonds and physical forces (such as functional group changes, hydrogen bonding, electron donor–acceptor, and electrostatic interaction) [21]. A number of factors, such as the surface chemistry and pore structure of the adsorbent, particle size, nature of the adsorbent, pH, temperature, and contact time, influence adsorption efficiency. The properties of the adsorbate, its molecular weight, structure, and size, as well as polarity, also have great significance [21,22,23].

The aim of this study is to use porous starch obtained by a three-step modification process (ultrasound, enzymatic hydrolysis, and freeze-drying) as a potential carrier for gallic acid, to describe the adsorption process, and to find those physicochemical properties described in detail in the manuscript (Part I) [24] that will have the most significant impact on this process. We hope that the wettability parameters of native and modified starches and also the adsorption studies carried out with gallic acid compounds will allow us to extend these characteristics to related substances, e.g., non-flavonoids or flavonoids in general.

## 2. Results and Discussion

### 2.1. Wettability and Surface Parameters of Starch before the Adsorption Process

Starch is a polar biopolymer, so it can be expected to have a higher ability to interact with polar substances, such as water and formamide, due to the interactions of dipoles and/or polar groups. Apolar diiodomethane CH_2_I_2_, due to the lowest affinity for starch and the lack of polar groups in the structure, should show the fastest penetration time. However, the matter is not that simple. The course of the wetting process is influenced by a number of other parameters; in addition to the porous structure and the size of the specific surface area, the size of the pores, their shape, the viscosity of the system, and surface tension may be decisive. This makes the process very complicated and multi-stage. However, it is certain that such a deep analysis of the wetting process is the starting point when discussing the adsorption process and can be very helpful in determining the adsorption mechanism in systems using native and modified starch.

**Figure 1:**
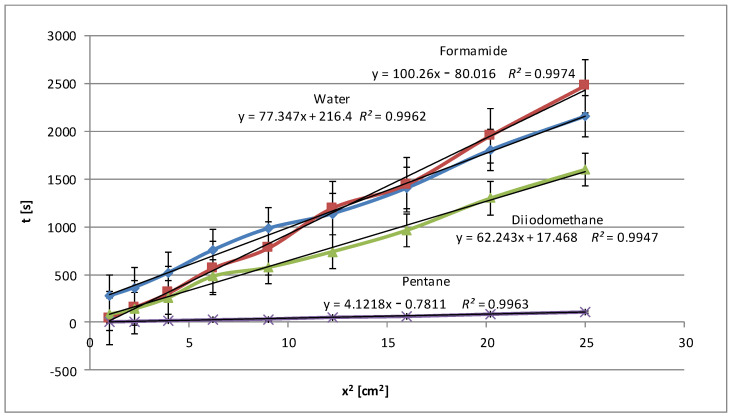
Wetting times of native potato starch as a function of the penetration square distance of the tested liquid (water—blue line, formamide—claret line, diiodomethane—green line, pentane—violet line).

Figure 1 shows the relationships obtained for native potato starch. In addition to the three test liquids (water, formamide, diiodomethane), the wetting process was also performed with pentane, as these data are needed to determine the effective capillary radius (details are described in the description of the method; see Section 3.2.1 and Section 3.2.2). Pentane, as an *n*-alkane, is used for these calculations due to its simple molecular structure and physicochemical properties, which define the surface properties well. The apolarity of pentane means its ability to wet polar substances such as starch is minimal or nil. This is reflected in the very low slope of its regression line. 

For this type of starch, the best matches were found both before and after modification above *R*^2^ *=* 0.99. Each graph contains standard deviations for individual measurements. High values of the determination coefficient *R*^2^, exceeding 0.99 for each of the tested test liquids, confirm that the linear regression model describes the relationship between the wetting time and the square of the penetration distance very well, indicating the linear nature of the Washburn equation.

In fact, the wetting time with polar water is shorter than the wetting time with less polar formamide, which may suggest that the interactions of the polar groups of HCONH_2_ with starch are more effective than water dipoles. This may also be due to the difference in the surface tension values of both liquids and different micropore penetration efficiency. It is also worth emphasizing that such a situation did not occur during the entire process. Approximately halfway through the process, the water showed longer penetration times, and then the situation reversed. It is possible that molecules can effectively fill small micropores. This process is supported by strong intermolecular interactions and the ability of molecules to form hydrogen bonds. In addition to the degree of polarity, other types of interactions, such as van der Waals forces or hydrophobic interactions, may be important. On the other hand, apolar diiodomethane shows fast penetration, which was proven to be accurate according to the graph presented. Only the results for pentane, which is understandable for an alkane compound with a very low surface tension (15.49 mN/m at 25 °C), were lower. 

**Figure 2:**
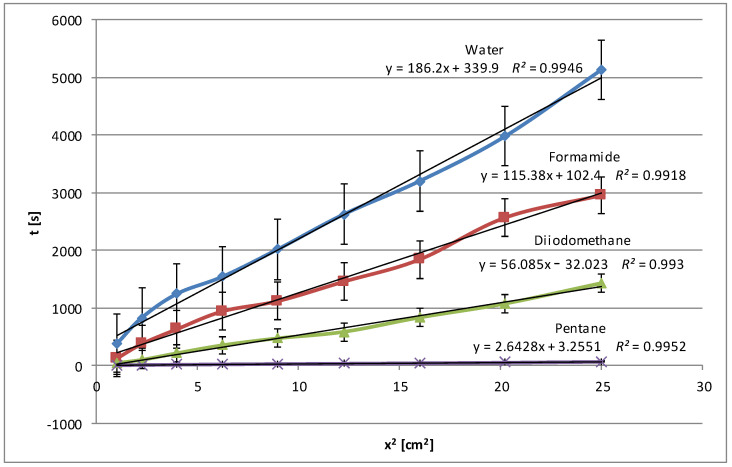
Wetting times of modified potato starch as a function of the penetration square distance of the tested liquid (water—blue line, formamide—claret line, diiodomethane—green line, pentane—violet line).

However, the correctness of the methodology was confirmed even though the plates were covered with a starch dispersion in laboratory conditions, which did not guarantee high homogeneity, unlike commercial plates. The presented linear relationships describe the relationship between the wetting time and the length of the starch penetration section quite well, demonstrating a high degree of fit of the model to the data according to the Washburn equation (Equation (1)). A fit of *R*^2^ value of 0.95 and above is considered sufficient in this method. Comparing Figure 1 and Figure 2, what is immediately noticeable is the doubling of the penetration times for water. Therefore, the range of the scale is double. In this case, penetration times also increased, but not so dramatically. The curves are clearly separated from each other, which may confirm the homogeneity of the examined layers. The higher results obtained for polar liquids and lower results for non-polar liquids (diiodomethane and pentane) confirm the increase in polarity in the case of modified potato starch. Water and formamide, due to their high polarity, are able to form hydrogen bonds with starch, which results in an increase in its penetration time.

In contrast to this process, diiodomethane and pentane, as apolar liquids, have weaker or no interactions with polar starch molecules; they do not penetrate deep into the starch but move along the surface, which is manifested by the highest wetting (in this case displacement) speed and the lowest times. 

**Figure 3:**
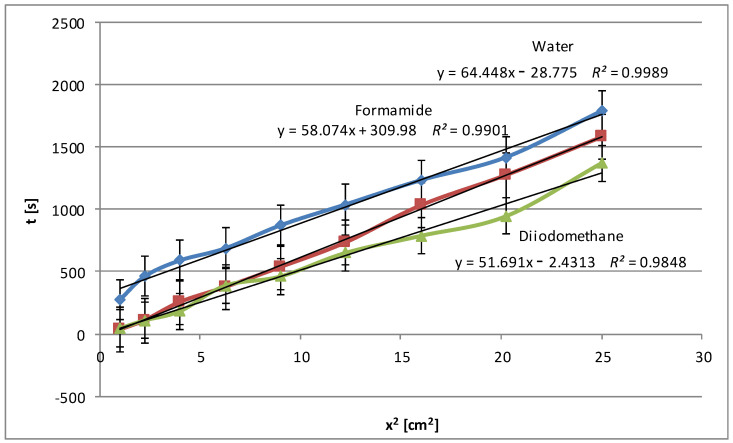
Wetting times of native pea starch as a function of the penetration square distance of the tested liquid (water—blue line, formamide—claret line, diiodomethane—green line).

The next figure (Figure 3) shows the wetting time dependencies obtained for pea starch. The low values of wetting times (below 2000 s) were obtained, and, what is more, the curves are close to each other for polar and non-polar liquids. This may suggest the weak polar nature of this kind of starch. This conclusion is also confirmed by the low zeta potential values described in detail in Part I [24], which indicate a small charge accumulated on the surface of the granules of this type of starch. As expected, water achieves the greatest penetration time, as seen from the trend line’s highest slope. Formamide is in the middle, while diiodomethane has the shortest penetration time. This is because the most polar water interacts strongly with polar starch, extending the time it takes to wet the starch surface. Apolar diiodomethane has the lowest degree of interaction with the starch. Formamide, which is less polar than water, falls between the two, presenting a medium rate of starch wetting. The obtained results are consistent with the adsorption data discussed in detail in Part I [24], i.e., for example, with the smallest specific surface areas. Again, high values of the coefficient of determination *R^2^* for all three liquids (water: 0.999, formamide: 0.990, diiodomethane: 0.985) indicate a very good fit of the linear model to the collected data.

**Figure 4:**
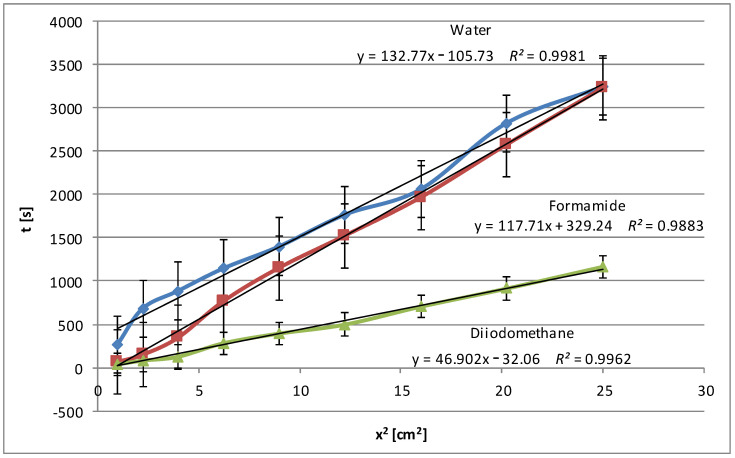
Wetting times of modified pea starch as a function of the penetration square distance of the tested liquid (water—blue line, formamide—claret line, diiodomethane—green line).

The modification of pea starch slightly increases its polarity, which causes water and formamide to penetrate the modified starch deeper than the native pea starch and therefore the wetting process is slower. Therefore, additional time is needed to create a network of hydrogen bonds (Figure 4). Diiodomethane, as an apolar liquid, does not form hydrogen bonds with starch. The increased polarity of the modified pea starch makes diiodomethane penetrate it faster, but only on the surface, because there is no resistance associated with the interaction of polar groups of starch with non-polar diiodomethane. In this case, bigger deviations from linearity are visible, especially for water. Such deviations in the penetration process may result from the manual coating of the plates, which may lead to surface inhomogeneities.

**Figure 5:**
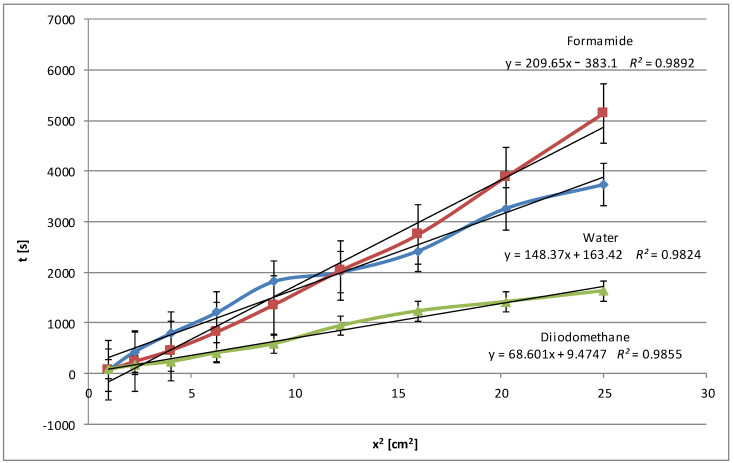
Wetting times of native corn starches as a function of the penetration square distance of the tested liquid (water—blue line, formamide—claret line, diiodomethane—green line).

Figure 5 shows the average values of the wetting rate of corn starch using water and diiodomethane. As mentioned above, the type and number of the functional groups in the structure of test liquids are essential, as they are responsible for the interactions with the polar groups of starch. In this case (Figure 5), similarly to potato starch (Figure 1), the relationships obtained for water and formamide are unclear over the entire penetration length. In the beginning, wetting times are longer for water, which translates into a slower rate, and the opposite for formamide. Near the halfway point, the situation reverses. However, such behavior of polar liquids and long values of wetting times indicate the polar nature of corn starch.

**Figure 6:**
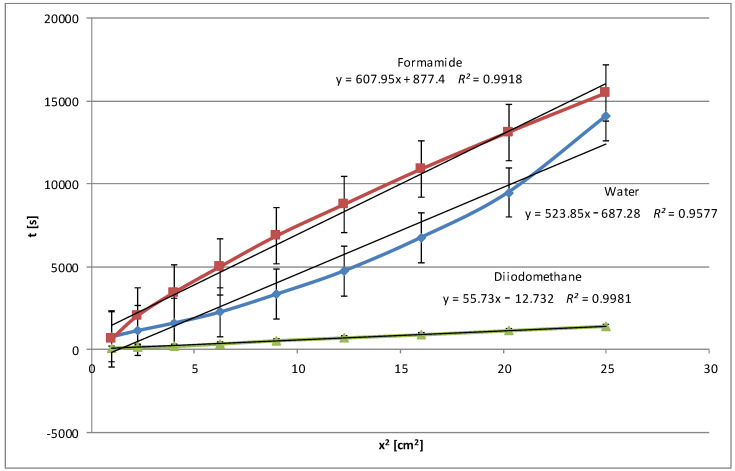
Wetting times of modified corn starch as a function of the penetration square distance of the tested liquid (water—blue line, formamide—claret line, diiodomethane—green line).

An analysis of all the wettability graphs shows that the liquid’s polarity directly impacts the wetting efficiency of native and modified starch. More polar liquids, such as water and formamide, effectively wet the starch by penetrating its structure with the possibility of forming polar bonds, while non-polar liquids, such as diiodomethane and pentane, move on the starch surface quickly or very quickly because the process takes place only on the surface and uses weak dispersion interactions. To confirm these conclusions, we present below the relationships obtained for modified corn starch (Figure 6). In this case, a significant increase in wetting times, almost 4-fold for water and 3-fold for formamide, was observed. This is understandable because the highest degree of hydrolysis, over 48%, was achieved for this starch, and the most significant changes in the specific surface area and electrokinetic zeta potential were noted, as described in Part I [24].

After the three-stage modification, the water wetting times of the plates covered with the modified starch dispersions were noticeably extended, sometimes even several times. This was the result of the presence of additional polar groups on the surface of the starch film due to hydrolysis, larger pore volume, and specific surface area. To obtain the full picture, similar tests were performed with polar formamide, non-polar diiodomethane, and pentane. In general, the wetting process of the modified starches with polar water and formamide was slower compared to the native starches. However, diiodomethane and pentane, which are non-polar liquids, penetrate modified starch faster than native starch. These changes suggest that the modification increases the polarity of all starches but to different degrees, increasing their interactions with polar liquids and decreasing them with apolar liquids. The relationships were expressed using linear equations with coefficients of determination *R*^2^, which in each case were very close to 1, which indicated an excellent fit of the linear model to the collected data.

The selection of liquids of different chemical natures (strongly polar, less polar, or apolar) gives an overview of the various types of interactions and enables a full description of the phenomenon of the wetting process. On this basis, the hydrophilic/hydrophobic nature of different kinds of starch can be determined, which consequently helps to select specific applications, e.g., adsorbents for food application [23], and will be helpful for a description of the gallic acid adsorption mechanism presented in the next Section 2.3.

### 2.2. Energetic Parameters of Starch before the Adsorption Process

It is well known that, apart from surface parameters, the energetic parameters of substances are also very important and influence the course of the adsorption process. The specific adsorption properties of starch are determined by the number of functional hydroxyl groups on the surface connected to the skeleton by covalent bonds, as well as the type of adsorption centers they create. These centers are considered as strong adsorption centers (unequal in terms of adsorption capacity) due to specific interactions with adsorbate molecules by the formation of hydrogen bonds or, more generally, donor–acceptor (acid–base) bonds. 

Knowing the number of surface groups allows the determination of the adsorption activity of starch. On the other hand, the concentration of surface polar groups is reflected in the surface free-energy components, which result from the type and size of intermolecular interactions. It is assumed that the amount of adsorption increases with an increase in the number of double bonds and polar functional groups present in the molecules of the substances penetrating the starch. The movement of the substances on the starch surface is primarily the result of the formation of hydrogen bonds between the functional groups of the substance and the active sites of starch. Depending on the type and structure of the test liquid, the adsorption process occurs or does not. Additionally, the adsorption mechanism itself may change as the concentration of the substance changes, so the interpretation of the results may be complicated.

In the case of modified starches, the situation may be completely different. The presence of free hydroxyl groups in native starch allows the introduction of a maximum of three substituents to each glucose unit. A distinguishing feature of this process is the concept of the degree of substitution (DS), which is defined as the number of functional groups introduced per one glucose unit. If the modifying factor is a multifunctional compound, one of its molecules may take part in the reactions with glucose units located in various starch chains. This is the so-called cross-linking reaction. 

During modification, native starch hydrolysis and oxidation processes may occur simultaneously, producing carboxyl and aldehyde ketone groups. The hydrolysis process may result in the substitution of hydroxyl groups, which changes or even reduces the hydrophilic properties, or the formation of new polar groups, e.g., carboxyl groups, as a result of which the hydrophilic character can increase. 

Below is a table (Table 1) presenting the adhesion tension values, ∆G (change in free energy (enthalpy) accompanying the replacement of the unit area of the solid–gas interface with the solid–liquid interface during the movement of the liquid in the porous layer) determined for all three types of starch before and after modification in contact with three test liquids of different polar natures (two polar and one apolar). In the test liquid–solid system, there may be not only a change in the surface tension of the solution but also changes in the solid–liquid interfacial tension.

As a result of adsorption, the hydrophilic surface of a solid may change its character to become less hydrophilic or even hydrophobic. The relationship between the amount of adsorbed substance at the phase boundary and the wetting parameters (e.g., contact angle) can be determined by changes in adhesive tension. The lower the value of the adhesive tension, the easier the wetting process takes place and, consequently, the adsorption process is facilitated. In the case of the adhesive tension for polar substances, this applies to the adsorption of hydrophilic substances, while in the case of apolar substances, this applies to hydrophobic substances. In the adsorption process of gallic acid, the relationships obtained for polar substances will be crucial.

The values calculated for water and formamide confirmed the polar nature of all types of starch: weak polar for pea starch and potato starch (medium values of adhesion tension) and the most polar for corn starch (the lowest value of adhesion tension). After modification, the polar character increased even more, especially in the case of corn starch, for which a more than 3-fold decrease in the value of adhesive tension for water and formamide was noted. In the case of the other two starches (potato and pea), this tension was about two times lower for water. The very high values obtained for diiodomethane (Table 1) can be extended to all hydrophobic substances. According to these values, it can be stated that adsorption of such substances is practically impossible on the starch surface, and after modification, these values increased even more, which further confirms this tendency. These considerations turned out to be helpful for description of the gallic acid adsorption mechanism on three types of native and modified starch presented in the next Section 2.3.

### 2.3. Adsorption of Gallic Acid on Native and Modified Starch

**Figure 7:**
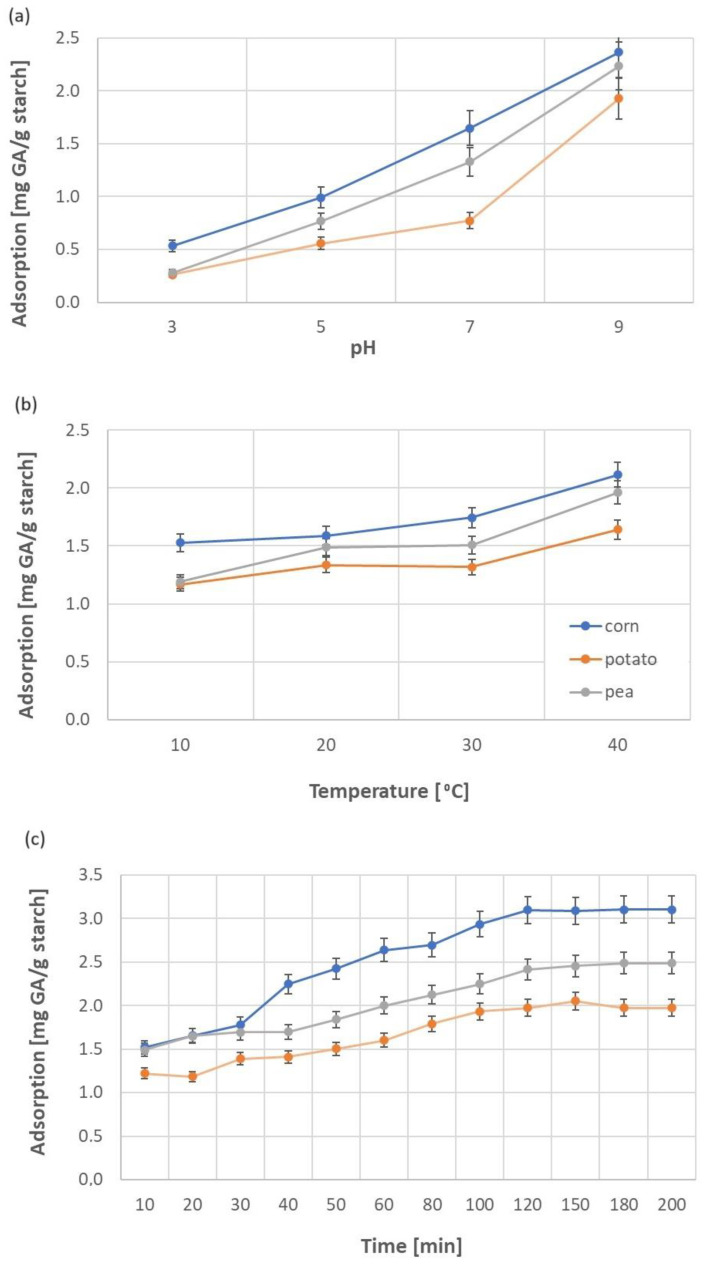
Effect of pH (**a**), temperature (**b**), and phase contact time (**c**) on the adsorption of gallic acid on native starches (corn—blue line, potato—orange line, pea—gray line).

Figure 7 shows the effect of pH, temperature, and phase contact time on the adsorption of gallic acid on native starch. In this particular case, we wanted to optimize the most important parameters affecting the adsorption of gallic acid on starch. Thus, we performed optimization only for the native starch, and then we adsorbed gallic acid on the modified starch, under identical conditions previously established for a given starch. In this way, we obtained a comparison of how the modification affected the sorption properties of starch. The highest amount of GA was adsorbed on corn starch for each parameter. In the pH range of 5–9, an increase in adsorption was observed with an increase in the pH of the reaction medium (Figure 7a). According to Ertan et al. [25] gallic acid interacts more intensely with biopolymers at a pH of 9 rather than at a pH of 7, as this compound converts to quinone, and crosslinking may occur. In our case, this process was also confirmed for all three starches. Between pH 7 and pH 9, a jump in the course of the relationship is visible. The largest increase in the trend line was noted in the case of potato starch (Figure 7a).

Similar experiments and results confirming the occurrence of these processes were described by Zhao and co-workers for cassava starch/chitosan/gallic acid systems obtained via pressurized fluid technology combined with ultrasonication [26]. The crosslinking between -COOH groups of gallic acid and -CH_2_OH groups of starch by ester bonds, and electrostatic interactions between COO^−^ and NH_3_^+^ were observed. These phenomena promoted film hydrophobicity by reducing the availability of -OH in starch, as observed by the decrease of the wettability parameter and as an effect of the decrease of water vapor permeability. These conclusions also confirm our considerations presented in Section 2.1 and Section 2.2 and data discussed in Part I [24]. Wettability and energy parameters are very sensitive to any changes occurring on the surface of starch granules (charge, number of hydroxyl groups, degree of substitution, etc.). Therefore, even a slight change may lead to a completely different course of the adsorption process, in this case of gallic acid.

The influence of temperature on GA adsorption was tested in the range of 10–40 °C (below the onset temperature of starch gelatinization; the range of T_0_ for potato, corn, and pea starches is 51–64 °C [27,28]). For each starch, an increase in temperature of 30 °C caused an approximately 1.3–1.5-fold increase in the amount of adsorbed GA (Figure 7b). Adsorption reached equilibrium after 120 min for corn starch, 150 min for potato starch, and 180 min for pea starch (Figure 7c). After these time periods, the highest amounts of adsorbed gallic acid were noted for corn starch at 3.10 mg/g, then for pea starch at 2.49 mg/g, and the lowest adsorption was recorded for potato starch at 2.05 mg/g. These results correlate well with the values of S_BET_ for modified starches (1.66 m^2^/g for corn, 0.40 m^2^/g for pea, and 0.22 m^2^/g for potato starch) obtained in Part I [24].

**Figure 8:**
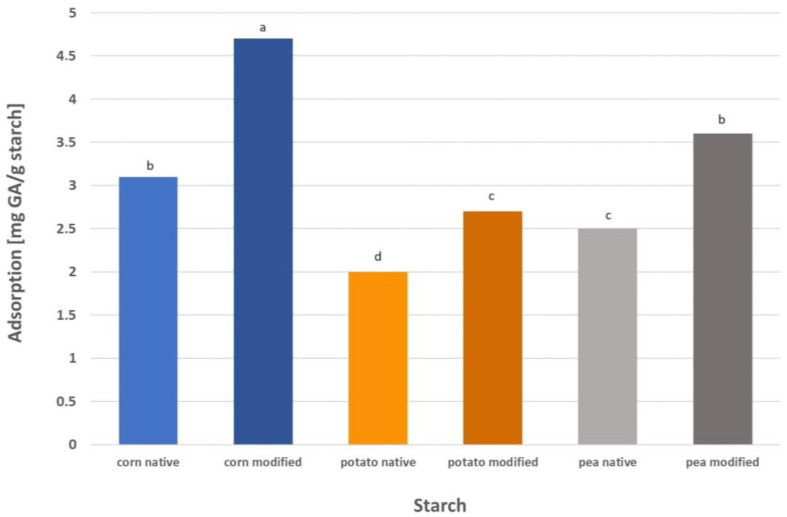
Adsorption of gallic acid on native and modified starch (means with different letters are significantly different, *p* < 0.05).

GA adsorption on modified starch was performed under the optimized conditions for native starch (pH 9, and 40 °C for all starches, and the phase contact time appropriate for a given starch), and the results are presented in Figure 8. The adsorption of GA on modified corn starch was 51% higher than on native starch. In the case of pea starch, the adsorption of this compound was 45% higher, and for potato starch it was 38% higher. After three-stage modification, the starch granules retained their structure and shape, but irregularities and pores were visible on the surface, especially in the case of corn starch [24]. It is well known that the surface morphology of the adsorbent affects its adsorption performance. The presence of irregularities and numerous pores on the surface of the modified starches influences their potential as adsorbents. Starch granules with pores demonstrate enhanced adsorption capacity compared with native starch granules as the content of hydroxyl groups increases. The adsorption performance of modified starch mainly depends on pore characteristics and, more precisely, on the specific surface area. The highest specific area was noted for corn starch (1.66 m^2^/g), which also showed the highest adsorption capacity toward gallic acid. Next in order were pea (0.40 m^2^/g) and potato (0.22 m^2^/g) starches, which correlates well with the results obtained for GA adsorption on these starches presented in Figure 8 [24]. Wang et al. [17] carried out the adsorption of grape seed proanthocyanidins on porous corn starch prepared by the enzymatic method using α-amylase and amyloglucosidase. The adsorption kinetics curve presented in these studies shows that after approximately 120 min, the adsorption curve flattened and equilibrium was achieved. Despite a slightly different modification methodology than in our case and a different adsorbed substance, the time to reach equilibrium for corn starch is identical, 120 min. This is a good future prognosis for the use of the starches tested by us for the adsorption and/or encapsulation of not only gallic acid but other substances with a similar structure or a similar adsorption mechanism. Wang et al. [17] reported that the amount of proanthocyanidins adsorbed on modified starch increased by approximately 10% compared to native starch. Similar results were obtained by Jiang et al. [15], who achieved adsorption equilibrium after approximately 100 min and the adsorption rate of procyanidins on porous rice starch increased by almost 45% compared to native starch.

### 2.4. ATR-FTIR Spectra

**Figure 9:**
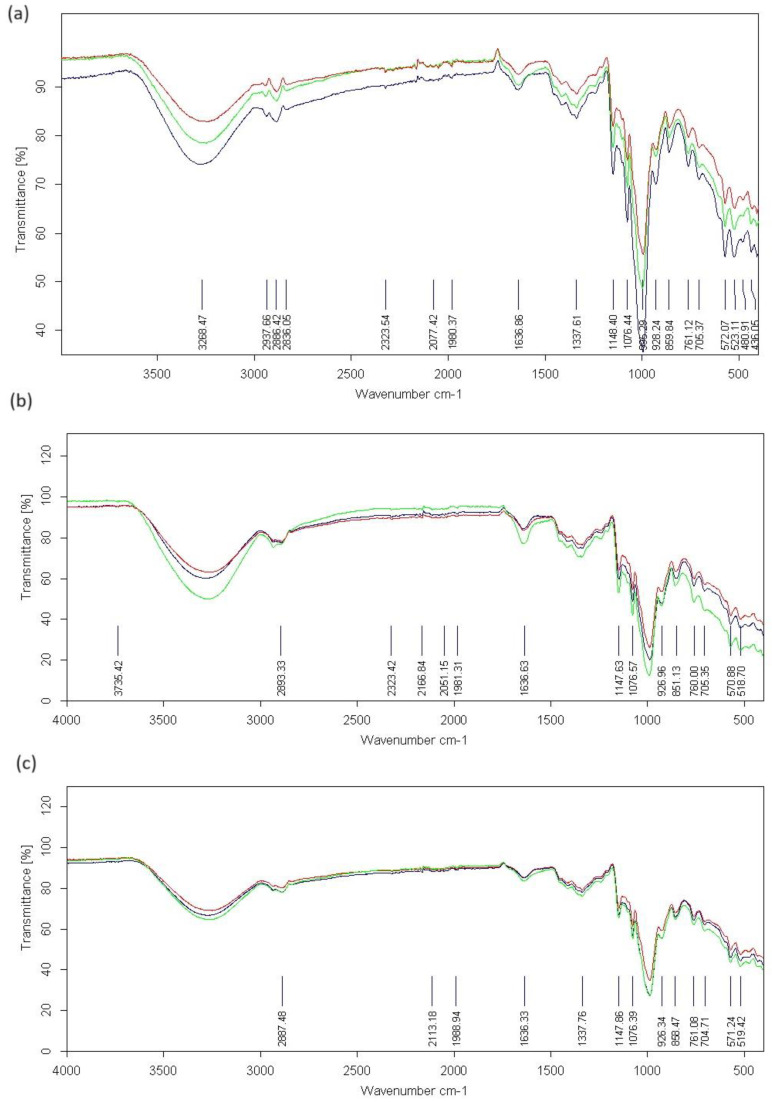
ATR-FTIR spectra of starches: native (red), modified (green), and modified complexed with gallic acid (blue). Types of starches: (**a**) corn starch, (**b**) potato starch, (**c**) pea starch.

As was expected, the positions of the characteristic absorption bands for modified starches and modified starches complexed with gallic acid in some cases differed from the positions of the bands for native starches, as shown in Figure 9. The intensity of the characteristic absorption bands of porous corn starch decreased compared to native starch. Scientists, in their studies, provide various explanations for this phenomenon. Jiang et al. [15] found that all characteristic absorption bands of native starch were present in the spectrum of porous starch, suggesting that the use of enzymatic hydrolysis did not change the chemical structure of obtained modified granules. However, according to Zhang et al. [29], the change in the intensity of the characteristic bands results from the fact that the formation of pores on the starch surface reduces the density of starch granules. We would be more inclined to accept the latter explanation, all the more so because slightly different results were observed in the other two starches. In the case of potato starch, a lower intensity of the characteristic modified starch bands was observed compared to native starch. In contrast, the intensity of the complexed starch bands increased compared to the modified ones. A similar relationship in the change in the intensity of the characteristic bands was observed in the case of pea starch.

After the adsorption process, the intensity of the bands of starch complexed with gallic acid decreased even more compared to the modified starch. The most considerable shift in the band position was observed in the case of pea starch for the band of starch complexed with gallic acid 3278 cm^−1^ compared to the native starch band of 3249 cm^−1^. Regarding another study, researchers performed the adsorption of gallic acid on porous starch and showed that the characteristic absorption bands of porous starch were not significantly changed after the adsorption of gallic acid, which was due to the fact that the adsorption process did not cause any changes in the molecular structure of the starch. Therefore, no new kind of chemical bonds were formed between gallic acid and the porous starch. The results suggested that gallic acid was mainly adsorbed by forming hydrogen bonds with porous starch [30]. Similar results were presented by Hu and Du [14], who reported no changes in the characteristic bands before and after adsorption of tea polyphenols on porous starch. They also found that adsorption occurred mainly through the formation of hydrogen bonds with functional groups on the microporous starch surface. In our research, we also consider this process as a main mechanism, e.g., the creation of new hydrogen bonds or replacing the old ones on the surface of the starch, in connection with the creation of a microporous structure. Since this microporous structure is most developed in the case of corn starch, the described mechanism most closely correlates with the results we obtained for this particular starch. Slightly different results were obtained for native pea and potato starch and also, after modification, again confirmed the lower polarity and lower porosity of these starches, which we have already discussed in this manuscript in Part I [24].

## 3. Materials and Methods

### 3.1. Materials

Modified starches (corn, potato, and pea purchased from Sigma-Aldrich Inc. (St. Louis, MO, USA), WPPZ S.A. (Luboń, Poland), and Roquette (Lestrem, France), respectively) were obtained by a three-step modification. Briefly, a 30% native starch suspension in distilled water was exposed to ultrasound (20 kHz, 170 W) for 15 min at 20 °C. The sample was centrifuged (5000 rpm for 5 min), and the sediment was washed with 96% ethanol and left to dry. After drying, the starch samples were used for enzymatic hydrolysis by an amyloglucosidase solution in a citrate buffer at a pH of 4.2. Hydrolysis was carried out at 50 °C for 24 h in a shaking incubator (150 rpm). After hydrolysis, starch granules were centrifuged and freeze-dried for three days at −45 °C. A detailed description of the modifications can be found in the first part of the manuscript, Part I [24]. Gallic acid was obtained from Sigma-Aldrich Inc. (St. Louis, MO, USA). All reagents used were of analytical grade purity.

### 3.2. Methods

#### 3.2.1. Wettability and Surface Parameters of Starch before the Adsorption Process

As mentioned in our previous paper [24], in addition to increasing porosity, the main goal of this research is to improve the antioxidant activity of starch through the use of gallic acid. It is well-known that phenolic acids exert antimicrobial activity beneficial to human health. During hydrolysis, the degradation of polysaccharides in starch to oligosaccharides is most probable, which can result in the higher solubility of the starch.

To check the wettability and surface properties for our three types of starch before and after modification, a thin-layer wicking method (TLC) was used for their dispersions deposited on glass plates analogous to our previous papers [31,32,33]. Briefly, 4 mL of starch dispersion (native or modified) prepared in water from the Milli-Q system was slowly spread using a pipette on glass plates until a uniform layer was obtained. The plates were then allowed to dry at room temperature and were next placed in a desiccator until measurements began. These studies were carried out using the Washburn procedure. To determine the course of the wetting process, it is necessary to know the surface tension of the solid and the solid–liquid interfacial tension. Both parameters cannot be determined directly for most systems, which is why determining the surface free energy of solids still poses many difficulties. In the case of powdered systems, such as tablets, soil minerals, and dyes, these parameters can be determined based on measurements of the rate of liquid penetration into the porous layer of this body using TLC. The relationship between the penetration time and the wettability of a solid was first determined by Washburn, who assumed that the movement of the liquid in the capillary occurs under the influence of capillary pressure. He proposed an equation describing the movement of liquid in a horizontal capillary and then showed that this equation is also true for a layer of powdered solid, in our case, a starch dispersion. Bartell, based on Laplace’s equation, stated that a powder film can be represented as a set of circular capillaries with a certain average radius. This is the radius of the equivalent capillary representing the pores in the powdered solid layer, called the effective radius of intergranular capillaries in the porous film R. The Washburn equation can be used if applied in the following form:

(1) $${\;x}^{2}=∆G\frac{Rt}{2\eta }$$

where R is the effective radius of the intergranular capillaries that are formed in the porous layer or column of a powdered solid, and ∆G is the change in free energy (enthalpy) accompanying the replacement of the unit area of the solid–gas interface with the solid–liquid interface during the movement of the liquid in the porous layer. Under constant pressure and temperature conditions for a series of plates for which the R parameter was experimentally determined, the plate wetting time t as a function of the square of penetration distance ${\;x}^{2}$ should be a straight line with a slope determined by ∆G.

The tested (probe) liquids are usually two polar (water) and one apolar (i.e., diiodomethane) liquids (see Table 2). The surface tension of the probe liquids was taken from van Oss and Good [34]. The calculation of the apparent surface free energy (SFE) and their changes provide remarkable information about even slight changes in energetic conditions, surface topography, and possible interactions occurring on the surface. Wettability parameters, especially by water, are also important due to the process of microorganism growth, which is crucial in food applications. 

According to van Oss et al. the total surface free energy of the examined surface is the sum of different types of interactions described by the Lifshitz-van der Waals ($\gamma_{s}^{LW}$) component as well as the electron–donor ($\gamma_{s}^{-}$) and electron–acceptor ($\gamma_{s}^{+}$) parameters [34,35]:

(2) $$W_{A}=\gamma_{L}(1+\cos\theta )=2\sqrt{\gamma_{S}^{LW}\gamma_{L}^{LW}}+2\sqrt{\gamma_{S}^{+}\gamma_{L}^{-}}+2\sqrt{\gamma_{S}^{-}\gamma_{L}^{+}}$$

where *W_A_* denotes the work of adhesion of a liquid to the solid surface, $\gamma^{LW}$ is the Lifshitz–van der Waals component, $\gamma^{-}$ is the electron–donor parameter, $\gamma^{+}$ is the electron–acceptor parameter, the symbol *S* denotes the solid and *L* the liquid.

There are many papers describing thin-layer wicking methodology applied to finely divided solids with a porous structure or also non-porous substances. The authors confirm that the methodology used does not affect the porous structure or has only a minimal impact. This was confirmed by profilometric and topographic studies [36,37,38,39].

#### 3.2.2. Determination of the Effective Radius of Intergranular Capillaries

The effective radius of intergranular capillaries of the tested substrate on glass plates was determined by averaging the radius obtained for pentane. Potato starch dispersions were used for this purpose, because in this case, the most repeatable results were obtained. Pentane, as an *n*-alkane, is used for these calculations due to its simple molecular structure and physicochemical properties, which define the surface properties well. The apolarity of pentane means that it has the minimal or no ability to wetting of polar substances. This is reflected in the very low slope of its regression line. The obtained value (7.068 × 10^−4^ cm) of the effective radius was used for further calculations for all types of starch.

#### 3.2.3. Conditions of Gallic Acid Adsorption on Starch

The following adsorption conditions were optimized: pH, temperature, and phase contact time. Starch suspensions at each stage of the experiment were shaken at 200 rpm, then centrifuged for 10 min at 3000 rpm, and the supernatant was collected. The absorbance of supernatants was measured at a wavelength of 260 nm against distilled water. The analytical wavelength was read from the UV absorption curve of gallic acid. The amount of adsorbed gallic acid was calculated based on a standard curve prepared for gallic acid concentrations in the range of 2–20 µg/mL (y = 0.0509x − 0.0086, *R*^2^ = 0.9996). Each assay was performed in duplicate.

The optimization of pH was carried out for values of this parameter of 3, 5, 7, and 9. Starch suspensions in a gallic acid solution (1%) were made in 100 mL conical flasks at the specific pH. Before the temperature was optimized, all suspensions were shaken at 30 °C for 1 h. Starch suspensions in a gallic acid solution (1%) with an optimized pH were prepared in 100 mL conical flasks. Then, the temperature of the suspensions was adjusted to 10 °C, 20 °C, 30 °C, and 40 °C, and the samples were shaken for 1 h. The phase contact time was optimized in the range of 10–200 min. Samples of the starch suspension (1%) with an optimized pH and temperature were shaken. Subsamples were taken every 10 min for absorbance measurement. Starch samples (native and modified) with gallic acid adsorbed under the optimized conditions were dried and stored in a desiccator at room temperature.

#### 3.2.4. ATR-FTIR Analysis of Starch–Gallic Acid Complexes

Mid-IR absorption spectra for native starch, modified starch, and modified starch-gallic acid complexes were obtained using attenuated total reflectance–Fourier transform infrared spectroscopy (ATR-FTIR) (Alpha II, Bruker Optics Inc., Billerica, MA, USA). Measurements were performed in damped total reflection mode using a Platinum ATR diamond crystal accessory. A pinch of the dried sample was placed directly on the crystal (with a contact surface diameter of 1.8 mm) and pressed against its surface. Spectra were collected by performing 36 spectral scans at a resolution of 4 cm^−1^ in the wavenumber range between 4000 and 400 cm^−1^. All spectral analyses were performed using OPUS 8.5 SP1 software (Bruker Optics Inc., Billerica, MA, USA).

### 3.3. Statistical Analysis

The obtained results were subjected to statistical analysis in the astatsa.com online program. One-way analysis of variance (ANOVA) was used to compare the results, and then the Tukey post hoc test was used to determine the significance of differences between the group mean values. Statistical hypotheses were verified at the significance level of *p* < 0.05.

## 4. Conclusions

The three-step modification of potato, pea, and corn starches increased their polarity, but to different degrees, thereby increasing their interactions with polar liquids and decreasing them with apolar liquids. Wettability relationships were expressed using linear equations with coefficients of determination *R^2^*, which in each case were very close to 1, indicating an excellent fit of the Washburn model to the collected data. The selection of liquids of different chemical natures provides an overview of various types of interactions and enables a full description of the wetting process. On this basis, the hydrophilic/hydrophobic nature of starch can be determined, which helped describe the mechanism of gallic acid adsorption. Polar liquids, such as water, effectively wet starch by penetrating its structure, possibly forming polar bonds, mainly hydrogen bonds, while non-polar liquids quickly move on the starch surface using weak dispersion interactions. This process was also related to the values of liquid surface tension, adhesive tension, and different micropore penetration efficiency. After modification, hydrolysis may result in the substitution of hydroxyl groups or the formation of new polar groups, e.g., carboxyl groups, as a result of which the hydrophilic/hydrophobic nature of starch changes.

Surface and energy parameters, adsorption experiments, as well as ATR-FTIR analysis confirmed that among the examined starches, the best adsorbent of gallic acid is corn starch, both in native and modified form. Based on all analyses, it was found that porosity and polarity have the greatest impact on the adsorption rate regarding the physicochemical parameters. After modification, the adsorption capacity of corn starch was 51% higher than its native counterpart. In the case of potato and pea starches, higher adsorption after modification was also observed as an effect of higher porosity. These results are consistent with the results of the surface determinations, porosity characterization, and zeta potential (charge) of all tested starches presented in Part I [24]. Overall, we hope that such adsorption studies conducted on gallic acid will be continued and can be extended to related substances (non-flavonoids or flavonoids).

## Figures and Tables

**Table 1:** Adhesive tension for water (W), formamide (F), or diiodomethane (D) in the wettability process of different kinds of starches.

Starch		Adhesive Tension W [mN/m]	Adhesive Tension F [mN/m]	Adhesive Tension D [mN/m]
**Corn**	**native**	16.26 ± 0.3	48.64 ± 0.6	113.22 ± 3.0
	**modified**	5.07 ± 0.1	14.70 ± 0.7	141.43 ± 1.0
**Potato**	**native**	29.28 ± 0.3	97.37 ± 0.4	124.08 ± 4.1
	**modified**	12.60 ± 0.6	79.16 ± 0.2	142.52 ± 2.5
**Pea**	**native**	39.79 ± 0.8	134.11 ± 0.1	151.37 ± 1.4
	**modified**	19.59 ± 0.6	72.21 ± 0.2	171.19 ± 1.2

**Table 2:** Surface tension of the probe liquids and its components used for wettability measurements in mN/m [35].

Probe Liquid	*γ^TOT^*	*γ^LW^*	*γ^+^*	*γ^−^*	*γ ^AB^*
**Water**	72.8	21.8	25.5	25.5	51
**Formamide**	58.0	39.0	2.28	39.6	19
**Diiodomethane**	50.8	50.8	0	0	0

## Data Availability

The data presented in this study are available on request from the corresponding authors.
